# Ultra-Fast Polarity Switching, Non-Radioactive Drift Tube for the Miniaturization of Drift-Time Ion Mobility Spectrometer

**DOI:** 10.3390/s22134866

**Published:** 2022-06-27

**Authors:** Lingfeng Li, Hao Gu, Yanzhen Lv, Yunjing Zhang, Xingli He, Peng Li

**Affiliations:** School of Electronic and Information Engineering, Soochow University, Suzhou 215006, China; lingfengli@suda.edu.cn (L.L.); hguguhao@stu.suda.edu.cn (H.G.); lvyanzhen110@gmail.com (Y.L.); yjzhang1223@suda.edu.cn (Y.Z.); hexingli@suda.edu.cn (X.H.)

**Keywords:** ion mobility spectrometer (IMS), polarity switching, non-radioactive

## Abstract

Drift-time ion mobility spectrometer (DT-IMS) is a promising technology for gas detection and analysis in the form of miniaturized instrument. Analytes may exist in the form of positively or negatively charged ions according to their chemical composition and ionization condition, and therefore require both polarity of electric field for the detection. In this work the polarity switching of a drift-time ion mobility spectrometer based on a direct current (DC) corona discharge ionization source was investigated, with novel solutions for both the control of ion shutter and the stabilization of aperture grid. The drift field is established by employing a switchable high voltage power supply and a serial of voltage regulator diode, with optocouplers to drive the ion shutter when the polarity is switched. The potential of aperture grid is stabilized during the polarity switching by the use of four diodes to avoid unnecessary charging cycle of the aperture grid capacitor. Based on the proposed techniques, the developed DT-IMS with 50 mm drift path is able to switch its polarity in 10 ms and acquire mobility spectrum after 10 ms of stabilization. Coupled with a thermal desorption sampler, limit of detection (LoD) of 0.1 ng was achieved for ketamine and TNT. Extra benefits include single calibration substance for both polarities and largely simplified pneumatic design, together with the reduction of second drift tube and its accessories. This work paved the way towards further miniaturization of DT-IMS without compromise of performance.

## 1. Introduction

DT-IMS, normally working under ambient pressure, is a sensitive technique for the detection of gas phase charged molecules based on their different mobility in the electric field [1]. It can either work as a standalone device [2,3,4] or in conjunction with other analytical instruments like Gas Chromatograph (GC) as its detector [5,6,7]. Its popularity largely attributed to the successful application in the detection of trace explosives, narcotics, Chemical Warfare Agents (CWA) and Toxic Industrial Compounds (TIC) [8,9,10,11]. According to the chemical composition the ionization process could result positive or negative ions, such as majority of nitro-based explosives are detected in the negative mode, and peroxide explosives including HMTD and TATP in the positive mode [12,13,14,15,16]. Other molecules can produce both positive and negative ions simultaneously such as methyl salicylate, for which measurement in both modes can further improve the accuracy of identification [17].

To characterize ions of both polarities, common methods is to use two drift tubes of each to be setup for one polarity, which clearly brings along increased volume, power consumption and system complexity [18,19,20,21,22,23]. An alternative way is to switch the polarity on a single drift tube to analyze positive and negative ions alternately, which is so called polarity switching DT-IMS. With this method it is an important that the switching is fast enough so that no information will be lost, especially when the concentration and identity of analyte vary over time. An example is the detection of TATP, which forms positive adduct ion and evaporates very quickly under the temperature condition suitable to thermally desorb explosives. If the switching of polarity takes a few seconds and the device happens to work under the negative mode when the TATP is sampled, the analyte will be gone when the polarity is switched back to positive mode, ending up a false negative result. In another application, when used as a detector for GC the switching also has to be fast as possible, since the elution peak only last for seconds and each measurement must take shorter time than the standard deviation to restore the gaussian peak with high fidelity [24].

To achieve fast switching of polarity, there are four major considerations for the design of the instrument. The first is to establish uniform electric filed in the drift tube after the change of voltage, which requires minimal capacitance between the electrodes used to define such electric field [24,25,26]. Another benefit for smaller capacitance is the reduction of power consumption, allowing the use of smaller power supply module for high fast switching instrument. The second is concerning the aperture grid in front of the faraday plate, which plays a key role for the elimination of mirror current effect on the ion detector [24,26]. The potential difference of aperture grid and faraday plate is defined by circuit in between, comprising a resistor and a capacitor connecting in parallel. The use of capacitor is to suppress the noise on the faraday plate. If its capacitance is too small then the circuit is prone to noise, and if too large the switching speed will be limited. The third is the design of ion shutter to meet the requirement of polarity switching [27]. And finally, when DC corona ionization source is used, extra complexity for the arrangement of electric field in the ionization/reaction region will be introduced to the system comparing to that of radioactive ionization source.

Zhou et al. reported the change of metal electrode from thin rings to thick ones and the switching recovery time was shortened from 20 s to 2 s, attributing to the reduced parasitic capacitance and charge accumulation, although 2 s seems still too long for the simultaneous detection of compound with different charge polarity [28]. Pershenkov et al. proposed to use an independent power supply for the aperture grid capacitor to reduce the discharge time, which requires extra cost and volume of the instrument [26]. Hitzemann et al. used a much smaller capacitor (220 pF) for the aperture grid, in combination with field switching ion shutter, to achieve a polarity switching time of 12 ms on a test system based on radioactive ionization source [24]. Although fast switching was achieved in this design, small aperture grid capacitor leads to the problem of noise control and faraday plate on HV end requires more sophisticated design of amplification circuit.

In this work, we have developed methods and implementation for an ultra-fast polarity switching DT-IMS based on DC corona discharge ionization. With a special design of driving circuit, only a single HV power supply is needed for both the ionization source and drift tube. For the ion shutter, a Tyndall-Powell (TP) ion gate was used for its simple structure, with the voltage change controlled by an optocoupler to synchronize with the polarity change of the system. A novel circuit was proposed to work with the aperture grid, eliminating charging cycle and therefore the need of extra power supply, yet allowing the use of large capacitor to filter the noise at same time. Meanwhile, with this implementation only one calibration substance is needed for both polarities, further reduced the complexity and overall volume. Coupled to a thermal desorption sampler and using narcotics and explosives as example analytes, the developed instrument was characterized for both switching speed and analytical performance.

## 2. Experiment

### 2.1. Instruments and Reagents

#### 2.1.1. Instruments

**Figure 1:**
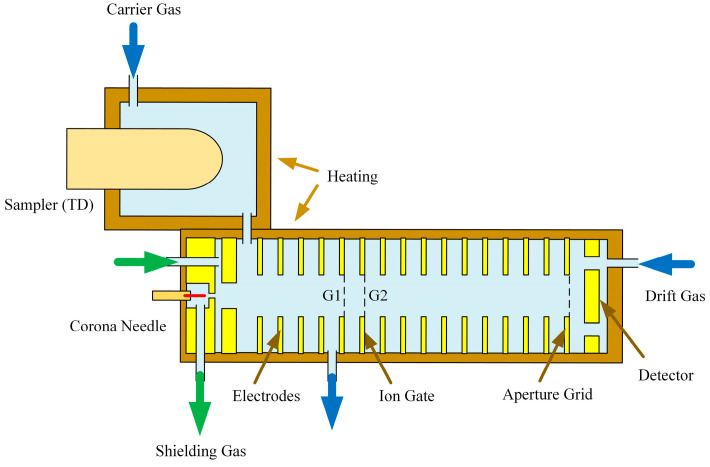
Structure illustration of the developed DT-IMS.

The DT-IMS system investigated is mainly comprised of sampler, ionization source, ion shutter, drift tube and ion detector (faraday plate), as shown in Figure 1. The DC corona discharge ionization source employs a needle-hole structure, with the distance between the needle and counter electrode set to be 2 mm, the corona discharge voltage is 2500 V. The counter electrode and G1 of ion shutter encompassed a reaction region with length of 25 mm and field strength of 30 V/mm. A TP ion gate is used as ion shutter with 0.5 mm as shutter width between G1 and G2, 0.18 ms as opening time, 30 V/mm as ion gate opening field strength, −60 V/mm as ion gate closing field strength, 50 mm as length of drift tube and 30 V/mm as field strength. The distance between G1 and the end of the reaction region is 1.17 mm, and the distance between G1 and the front of the drift tube is also 1.17 mm. The ring electrodes used for both reaction region and drift tube are 16 mm for inner diameter, 1.37 mm for thickness. The insulator rings are 1.17 mm for thickness and 18 mm for inner diameter, with slightly larger inner diameter to reduce the parasitic capacitance and also the charge accumulation on the surface. The end of the drift tube is an aperture grid, which has a distance of 1 mm from the faraday plate and an aperture voltage of 60 V. Confirmed by the measurement results, with this structure design the parasitic capacitance of the drift tube is reduced to 11.5 pF. The working temperature of the drift tube was set to 120 °C to comply with the 200 °C working temperature of the thermal desorption sampler for the detection of explosives and narcotics with low volatility. The gas used in the system is clean air filtered by active charcoal and 13x molecular sieve for both sample stream at 150 mL/min and drift flow at 200 mL/min. The dew point after purification is below −60 °C. To maintain the stability of discharge and consistency of ionization products, another gas flow of 700 mL/min is introduced as protection for the corona discharge ionization source.

A commercially available high voltage power supply module (Shanghai Taizhu, SMO series) is used to switch the polarity of applied voltage, with output voltage of ±6000 V and polarity switch time of 10 ms. The ion current is converted to voltage signal by a homemade transimpedance amplifier with bandwidth of 8 kHz and gain of 5G V/A. The mobility spectrums are collected through a data acquisition card (National Instruments, USB6210) with sampling rate of 100 kHz. The signal for polarity changes and operation of ion shutter are also provided by the output of data acquisition card.

**Figure 2:**
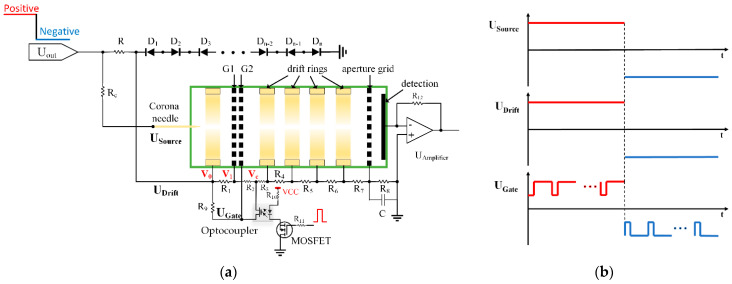
(**a**) Illustration of electric circuit; (**b**) time sequence of applied voltages.

Illustration of electric circuit and time sequence of driving signal are shown in Figure 2a,b, respectively. For the polarity switching of DT-IMS there are 3 applied voltages need to be reversed, including the ion source (U_source_), the drift tube (U_drift_) and G2 of the TP ion gate (U_gate_). Normally DT-IMS with corona discharge ionization source requires at least 2 HV modules to serve the ionization source and drift tube separately. In order to simplify the system for further miniaturization, in this work a single HV module was used for the entire system, with applied voltage and time sequence as shown in Figure 2b for U_source_, U_drift_ and U_gate_, respectively.

To implement the voltage settings with time sequence described in Figure 2b with a single HV supplier, a novel electronic circuit was designed as shown in Figure 2a. The end electrode of drift tube is connected to common ground so that the faraday plate is also around the ground, which reduces the critical requirements for signal amplification circuit. Apart from the resistors in serial connection as HV dividing network, a certain number of Zener diodes are inserted between the counter electrode of the ionization source and the ground to define the drift field and also avoid voltage drift of HV module. In this implementation 25 Zener diodes (Nexperia, BZB100) are used for ±2500 V voltage drop across the drift region, with a protection resistor R of 100 MΩ connecting to the HV output. The corona needle is connected to HV output through a large resistor R_c_ of 1 GΩ for limiting the discharge current, to get the discharge voltage towards the counter electrode.

The TP ion gate blocks the ions flux by setting up a reverse electric field between G1 and G2, with its driving circuit shown in Figure 2a. The trigger signal is applied on the MOSFET (PMV28ENEA) to control the open/close status of the ion shutter. An optocoupler (Infineon, PVA3055NPBF) is used to directly regulate the voltage of G2, so that the low voltage control signal can be separated from the HV. The voltages on both sides of the optocoupler are taken from the HV dividing network. When the optocoupler is closed, |Vc| is always smaller than |V1|, and therefore the electric field between G1 and G2 is in reverse direction to the drift region, the ion shutter is closed. On the other hand, when the optocoupler is open, exactly opposite situation is expected and the ion shutter is open. In this way the operation of the ion shutter worked with both polarities, with clear advantages over traditional half-bridge circuit, which cannot deal with the polarity switching due to the limitation that the gate volage has to be higher the drain voltage.

**Figure 3:**
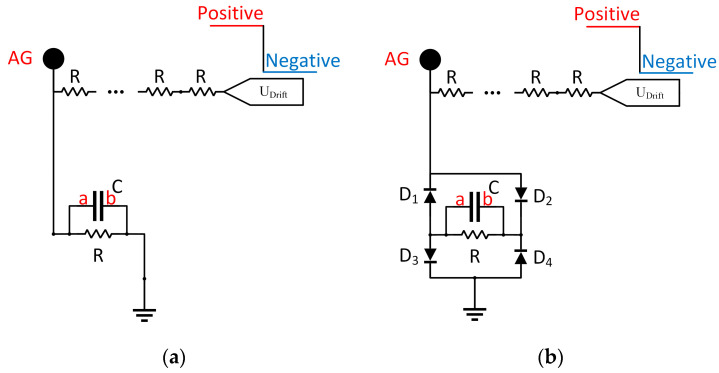
Aperture grid circuit: (**a**) traditional configuration; (**b**) this study.

The purpose of aperture gird in IMS instrument is to prevent the generation of induced current as the ion swarm approaches the detector, so that the voltage stability of aperture grid is of key importance for the quality of signal measured on the detector [24]. Normally a large capacitor C, which capacitance value is between 100 nF and 1 μF, is arranged between the aperture grid and ground, in parallel to the voltage dividing resistor R, to stabilized the potential difference between the aperture grid and the ion detector and hence reduce the noise, as shown in Figure 3a. But when the polarity is switched, the charging/discharging of this large capacitor will largely slow down the establishment of desired voltage on the aperture grid. Here in this work, we use 4 switching diodes (BAV21W) to avoid the charging/discharging of the capacitor and yet to maintain a stable voltage on the aperture grid, as shown in Figure 3b. The circuit works as the following steps: when U_drift_ is at +HV, diode D2&D3 will be on and D1&D4 are off, so that with capacitor C node a is with ground and node b is with positive, the electric field within the capacitor is from b to a; when the polarity is switched and U_drift_ is at -HV, D2&D3 will be off and D1&D4 are on, node a is with negative voltage and node b is with ground, the electric field within the capacitor is still from b to a. Therefore, with this circuit the voltage of aperture grid can be established to the desired value without the charging/discharge of the capacitor, which is a key factor to realized ultra-fast polarity switching of the system. In the following sections the performance of this circuit was compared and analyzed against the traditional circuit.

#### 2.1.2. Reagents

HPLC grade acetonitrile was purchased from Tedia Co Ltd. (Fairfield, CA, USA). Standard explosive samples of TNT, PETN, HMX, RDX, HMTD and TATP with con-centration of 1000 ng/μL were purchased from Beijing Shiji ‘aoke Biotechnology Co., Ltd. (Beijing, China). Standard narcotic samples of cocaine, ketamine, methamphetamine, heroin, morphine, and tetrahydrocannabinol with concentration of 1000 ng/μL were purchased from Shanghai Yuansi Standard Science and Technology Co., Ltd. (Shanghai, China). Molecular sieve 13x and activated charcoal were purchased from Sinopharm Chemical Reagent Co., Ltd. (Shanghai, China). Disposable Nomex swab was supplied by Suzhou Chuanche Specialty Materials Co., Ltd. (Suzhou, China).

### 2.2. Experimental Methods

#### 2.2.1. Preparation of Standard Solution

Analyte solutions of explosives and narcotics were prepared by stepwise dilution of 1000 ng/μL standard solution to 100 ng/μL, 10 ng/μL, 5 ng/μL, 1 ng/μL and 0.1 ng/μL.

Mixture samples were prepared following these steps: the 1000 ng/μL standard solution was firstly diluted by 10 times to 100 ng/μL, the diluted solutions of chosen analytes were then mixed together with 100 μL of each, and finally the mixed solution was made up to 1 mL using acetonitrile to get 10 ng/μL concentration for each analyte.

#### 2.2.2. Sampling Methods

For sample introduction 1 μL of sample solution was deposited on Nomex swab, which was then inserted into the sampling inlet of the thermal desorption unit after the solvent evaporated. The vapor of the analyte was then introduced into the DT-IMS system by the sample carrier gas flow. For each sample the test was repeated for at least 3 time to check the reproducibility.

## 3. Results and Discussion

### 3.1. Performance Evaluation of Polarity Switching DT-IMS

#### 3.1.1. Comparison of Aperture Grid Circuits

**Figure 4:**
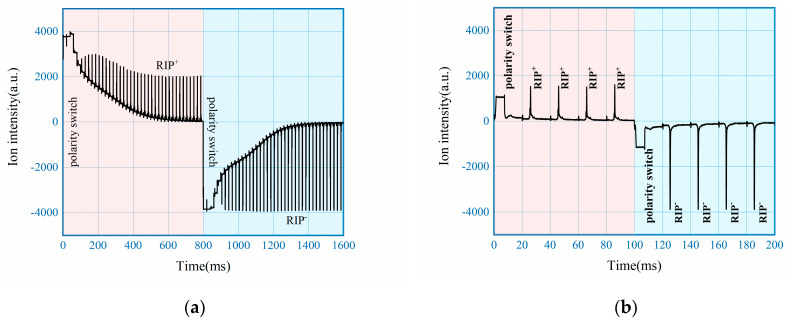
Aperture grid circuit: (**a**) traditional configuration; (**b**) this study.

Measured spectrums are shown in Figure 4, in comparison between the traditional aperture grid circuit and solution of this work. In both cases only Reactant Ion Peak (RIP) was measured without sample introduction, with the ion shutter opened once every 20 ms and synchronized with the polarity switching. With traditional aperture grid circuit as shown in Figure 3a, the positive mode of IMS is maintained for 800 ms before switching to negative mode, with 1600 ms of data collected as shown in Figure 4a. From the results it can be observed that after the switching of HV which took 10 ms, the baseline needs around 600 ms to stabilize for valid measurement. With the proposed circuit as shown in Figure 3b, measured spectrum using 100 ms as switching interval are shown in Figure 4b. From the results, it only took 10 ms to get stable spectrum on top of another 10 ms for switching, demonstrating around 30 times improvement for the speed of polarity switching with the new approach.

#### 3.1.2. Mobility Spectrum with Fast Switching

**Figure 5:**
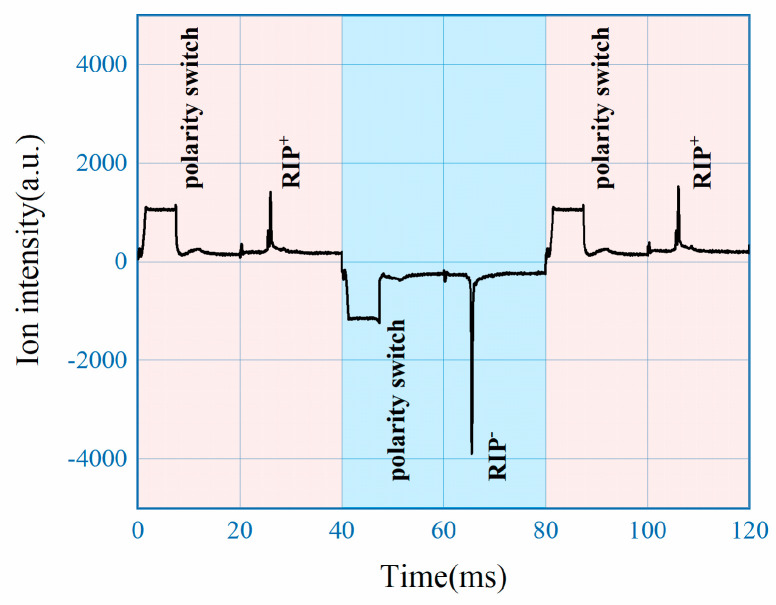
Effect of fast switching on the spectrum.

A consecutive measurement of 40 ms of positive mode(red), 40 ms of negative mode(blue) and another 40 ms of positive mode(red) is shown in Figure 5. The experiment setting is similar, that only RIP was collected with 20 ms interval for ion shutter and synchronized polarity switch of 40 ms. It can be observed from the results that for the first opening of ion shutter after the change of polarity, baseline restoration and overload of the amplifier due to the HV switch exist, which made the collection of data invalid, as marked on the plot. However, for the second opening of ion shutter after switching the baseline is fully restored with expected RIP, as shown in Figure 5. Switching back to positive mode at 80 ms results identical spectrum as that of 0–40 ms, meaning the developed DT-IMS can work continuously with the ability to acquire full spectrum including both polarity within 80 ms.

**Figure 6:**
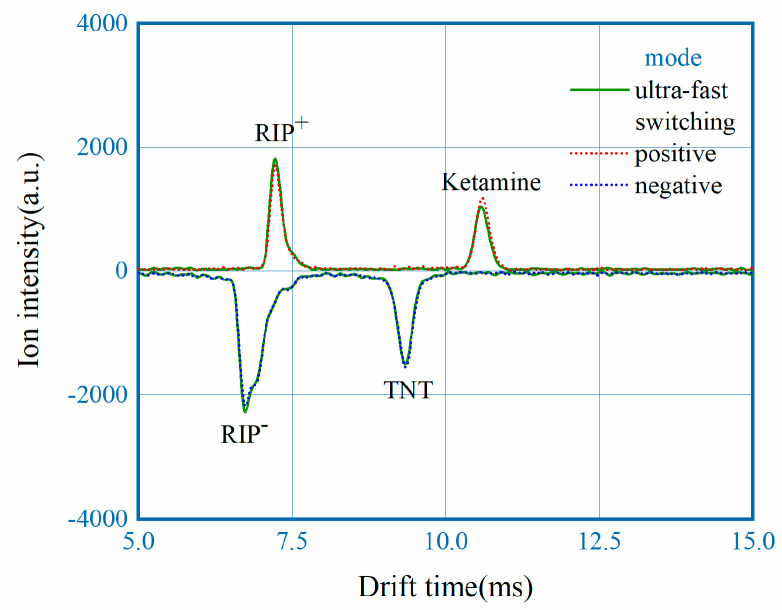
Spectrum in comparison, measured under ultra-fast switching mode, positive mode and negative mode.

To investigate the effect of polarity switching, measured spectrum with fast switching mode of 80 ms interval are put in comparison with results collected with single polarity under the same experimental conditions. For all three measurements, 5 ng of ketamine and TNT were used as analyte and the spectrum represented were all baseline corrected. From Figure 6 it can be observed that the drift time and intensity of both RIP and Product Ion Peak (PIP) are nearly identical, with maximum difference of peak intensity less than 3%. The results here demonstrates that fast polarity switching of the DT-IMS instrument develop in this work didn’t compromise the analytical performance.

### 3.2. Polarity Switching DT-IMS for the Detection of Explosives and Narcotics

#### 3.2.1. Effect of Switching Speed with Evaporated Samples

**Figure 7:**
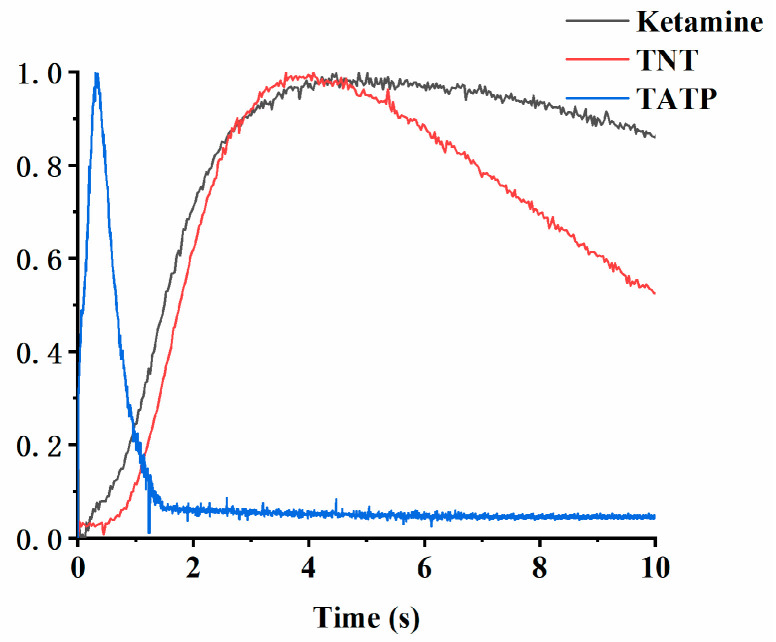
Concentration profile over thermal desorption time of ketamine, TNT and TATP.

It is well known that detection of narcotics and explosives with IMS requires both polarities, since both positive and negative product ions are generated during the ionization process. Due to various volatility, typical sample introduction method is thermal desorption, with relatively high temperature to cover analytes with high boiling point such as HMX. The concentration of vapor phase analyte hence varied over time as shown in Figure 7 for 3 typical samples including ketamine, TNT and TATP. The measurements were carried out on the developed DT-IMS instrument in single polarity mode (positive mode for ketamine of 10 ng and TATP of 100 ng; negative mode for TNT of 10 ng) with 20 ms data collection interval and 200 °C for thermal desorption. All data was normalized for comparison, with the vertical axis representing relative concentration. It can be seen that for given conditions, the maximum concentration appears in large time window, from less than 0.5 s for TATP to around 5 s for TNT and ketamine.

**Figure 8:**
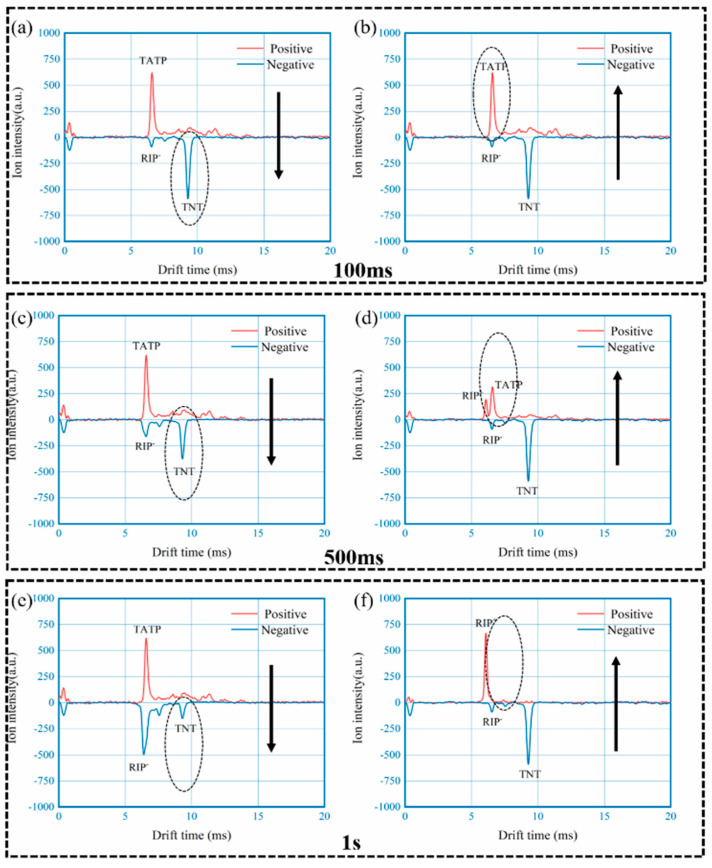
Effect of polarity switching time, mixture of 100 ng TATP and 10 ng TNT used as analyte: (**a**,**b**) for 100 ms; (**c**,**d**) for 500 ms; (**e**,**f**) for 1 s.

To examine the effect of polarity switching, an experiment was designed for the measurement of mixed sample comprising 100 ng of TATP and 10 ng of TNT, using the developed DT-IMS instrument with different time and switching direction settings, as shown in Figure 8. Spectrums of three different switching period, 100 ms (a and b), 500 ms (c and d) and 1 s (e and f), were taken with both directions of switching including positive to negative (a, c and e) and negative to positive (b, d and f). From the results it can be observed that when the switching is fast enough, such as 100 ms or 10 Hz in this case, it doesn’t matter how the polarity is switched, analytes from both positive and negative modes can be detected with negligible difference. However, as the polarity switching got slower the results are substantially different until analyte from the second polarity mode cannot be detect with 1 s switching period. In combination of the concentration profile shown in Figure 7, it could be concluded that the variation of chemical information, including identity and concentration of analytes, can only be accurately captured by single drift tube DT-IMS with fast polarity switching capability.

#### 3.2.2. Detection of Narcotics and Explosives

**Figure 9:**
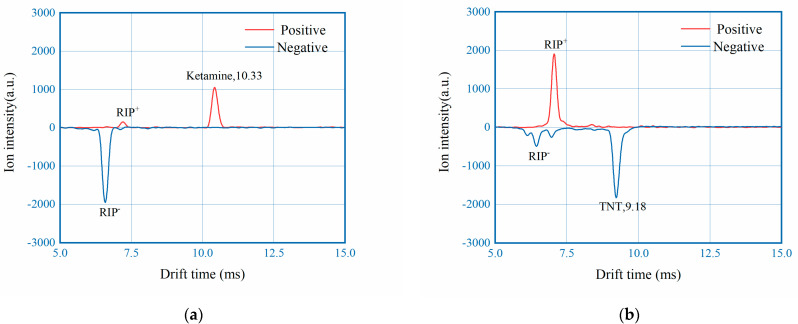
Example IMS spectrums: (**a**) 10 ng ketamine; (**b**) 10 ng TNT.

To further evaluate the instrument samples of six narcotics and seven explosives have been tested with switching time of 100 ms, example spectrums of 10 ng ketamine and 10 ng TNT are shown in Figure 9. Spectrums of other analytes can be found in Figures S1 and S2 of the supporting information.

#### 3.2.3. Validation of Calibration Method

For traditional DT-IMS with separate drift tubes, normally two calibration substances are needed to compensate the variation and inconsistency of the drift field, since the variation of voltage is independent for each polarity. However, with the polarity switching DT-IMS developed in this work, because the voltage across the drift tube is defined by the Zener diode for both polarities, in principle it is possible to use a single calibration substance.

Validation of the hypnosis was carried out with experiments using niacinamide, a common positive mode calibrator for IMS instrument with well recognized mobility, to calculate the reduced mobility of narcotic and explosive samples in comparison with the values reported in the literature [29,30]. The reduced mobility of niacinamide was reported to be 1.860 cm^2^·V^−1^·s^−1^, so in the system with niacinamide as calibrator, reduced mobility of other sample in the same mode can be calculated as:

(1) $$K_{0}^{\prime }\;=\;\frac{K_{0}\cdot t}{t^{\prime }}$$

where $K_{0}^{\prime }$ is the reduced mobility of analyte of concern, K_0_ is the reduced mobility of the calibrator, t′ and t are the drift time for analyte and calibrator, respectively. Spectrum data of narcotic and explosive samples from Section 3.2.2 was used to calculate the reduced mobility, which was summarized in Table 1 and Table 2.

As shown in Table 1 and Table 2, majority of the analytes tested demonstrated good consistency of measured K_0_ to that reported in the literature, with HMTD as the only exception for reasons to be further investigated [31,32,33,34,35].This result, the successful mobility correction of both polarities using a single calibrator with only positively charged product ion, proves the feasibility of not only to reduce a drift tube from the system, but also to eliminate the need for the second calibrator with associated container and supporting accessories, which is a substantial step for further miniaturization of the DT-IMS instrument. The underlying reason can be ascribed to the stable and consistent drift field of both working mode. In the developed system the voltage across the drift tube is defined by the bidirectional voltage regulator diodes for both modes, and since a single drift tube was used there will be no size inconsistency either from the manufacturing or elongated working, therefore the electric field for ion drifting will be strictly the same for either polarity, enabling mobility correction with a single calibrator.

#### 3.2.4. Detection of Mixed Samples

**Figure 10:**
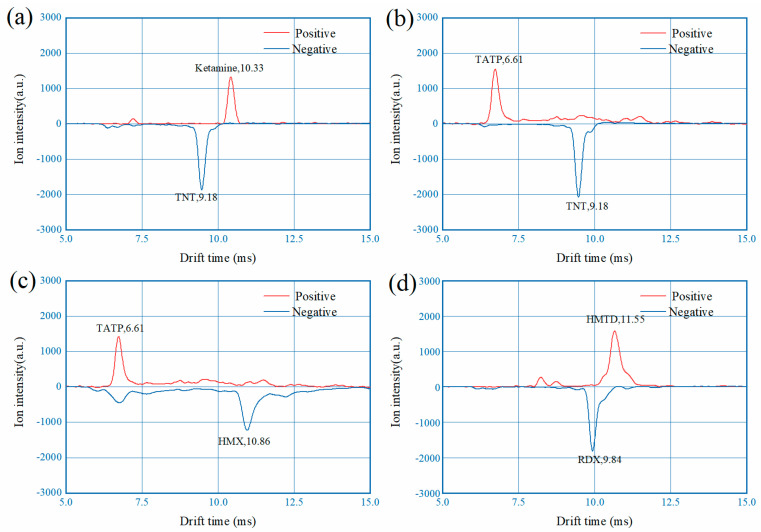
Measured spectrums of mixed samples: (**a**) 10 ng TNT and 10 ng ketamine; (**b**) 10 ng TNT and 100 ng TATP; (**c**) 100 ng HMX and 100 ng TATP; (**d**) 10 ng RDX and 100 ng HMTD.

With the hardware performance established such as switching speed and mobility accuracy, the polarity switching DT-IMS is tested with mixed samples of narcotics and explosives for the examination of detection capability. The mixed samples are prepared as described in the experiment section and tested with 100 ms switching time, as shown in Figure 10. Each prepared mixed sample intentionally include analytes of both polarities for demonstration. From the results it can been seen that with the current instrument all of the narcotic and explosive we had can be successfully detected, regardless of polarity and volatility.

#### 3.2.5. Limit of Detection

**Figure 11:**
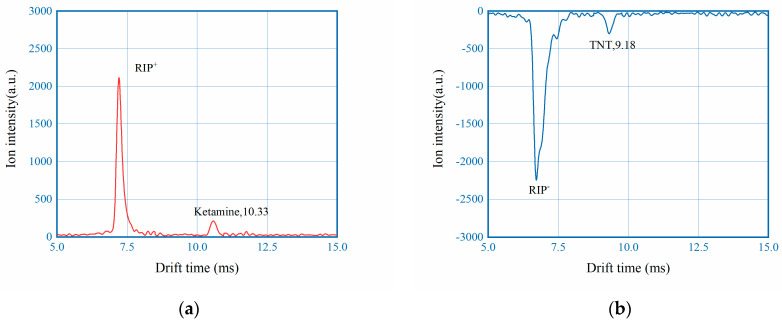
Example IMS spectrums of (**a**) 0.1 ng ketamine; (**b**) 0.1 ng TNT.

LoD of the developed instrument is also investigated with the standard of signal-to-noise ratio greater than 3. Commercial instruments normally claim LoD of 1 ng or even lower [29]. In this work two representative analytes, ketamine and TNT, are chosen to characterize the LoD by stepwise dilution. As shown in Figure 11 for both analytes 0.1 ng can be reliably detected.

## 4. Conclusions

In this work an ultra-fast polarity switching IMS drift tube with non-radioactive ionization source was designed and implemented, employing novel methods on the electronic design to switch the aperture grid and ion shutter. Stable spectrum can be measured within 20 ms after the change of polarity. Testing results demonstrated that the developed instrument can effectively detect common narcotics and explosives regardless of ion polarity and volatility. The LoD was lower than 0.1 ng and only a single calibrator was needed for mobility correction for both positive and negative modes. The proposed electronics and system design paved the path for the future miniaturization of DT-IMS, which can either be developed into a miniaturized hand-hold instrument, or a sensor to be used for GC and other separation technologies.

Some limitations of this work still exist:

1. The delay of optocoupler which was used to control the TP ion gate could possibly introduce systematic errors for the measurement of mobility.
2. The power consumption of the DC corona discharge ionization source is relatively large.
3. The integration of the system needs to be improved.

The future work we have planned includes optimization of the ion gate, replacing DC corona ion source with AC, and integration of not only the drift tube but also other peripherals such as flow control components etc.

## Figures and Tables

**Table 1:** Reduced mobility of 6 common narcotics.

No.	Sample	Molecular	Generated Ion	Drift Time/ms		K_0_/cm^2^·V^−1^·s^−1^ (Measured)		K_0_/cm^2^·V^−1^·s^−1^ (Literature [31,32,33])
				Average	Standard Deviation	Average	Standard Deviation	
1	Cocaine	C_17_H_21_NO_4_	[M+H]^+^	11.90	0.025	1.170	0.003	1.16
2	Ketamine	C_13_H_16_NOCl	[M+H]^+^	10.34	0.010	1.348	0.002	1.31
3	Methamphetamine	C_10_H_15_N	[M+H]^+^	9.05	0.020	1.536	0.007	1.601
4	Heroin	C_21_H_23_NO_5_	[M+H−CH_3_COOH]^+^	11.91	0.020	1.169	0.002	1.17
			[M+H]^+^	13.58	0.265	1.029	0.008	1.04
5	Morphine	C_17_H_19_NO_3_	[M+H−H_2_O]^+^	10.82	0.124	1.253	0.027	1.26
			[M+H]^+^	11.88	0.025	1.172	0.002	1.22
6	Marijuana	C_21_H_30_O_2_	[M+H]^+^	13.16	0.012	1.059	0	1.07

**Table 2:** Reduced mobility of 7 common explosives.

No.	Sample	Molecular	Generated Ion	Drift Time/ms		K_0_/cm^2^·V^−1^·s^−1^ (Measured)		K_0_/cm^2^·V^−1^·s^−1^ (Literature [31,34,35])
				Average	Standard Deviation	Average	Standard Deviation	
1	TNT	C_7_H_5_N_3_O_6_	[M−H]^−^	9.18	0.006	1.519	0.001	1.53
2	Black Powder	S	[S_3_]^−^	6.47	0.010	2.147	0.006	2.26
3	PETN	C_5_H_8_N_4_O	[M+NO_3_]^−^	11.90	0.012	1.171	0.002	1.19
4	HMX	C_4_H_8_N_8_O_8_	[M+NO_2_]^−^	10.86	0.010	1.282	0.002	1.28
5	RDX	C_3_H_6_N_6_O_6_	[M+NO_2_]^−^	9.84	0.020	1.415	0.003	1.42
6	HMTD	(CH_2_)_6_N_2_(O_2_)_3_	[M+H]^+^	11.38	0.150	1.225	0.016	1.55
7	TATP	C_9_H_18_O_6_	[(CH_3_)_2_C(O)OO]H^+^	6.60	0.012	2.120	0.023	2.15

## Data Availability

The data presented in this work are available in the article and Supplementary Materials.

## Supplementary Materials

The following supporting information can be downloaded at: https://www.mdpi.com/article/10.3390/s22134866/s1, Figure S1: Photos of the drift tube device; Figure S2: Example IMS spectrums of 5 narcotics: (a) 10 ng cocaine; (b) 50 ng methamphetamine; (c) 50 ng heroin; (d) 50 ng morphine; (f) 50 ng marijuana; Figure S3: Example IMS spectrums of 6 explosives: (a) 50 ng black powder; (b) 50 ng PETN; (c) 100 ng HMX; (d) 50 ng RDX; (f) 100 ng HMTD; (g) 100 ng TATP.
