# Supplementary material for: Ultra-Fast Polarity Switching, Non-Radioactive Drift Tube for the Miniaturization of Drift-Time Ion Mobility Spectrometer
Source: Sensors (Basel). 2022 Jun 27;22(13):4866. doi: 10.3390/s22134866 (PMC9269308; doi:10.3390/s22134866)
Supplement: Supplementary file 1 [file sensors-22-04866-s001.zip › sensors-1778916-supplementary.pdf]

Supporting Information

Ultra-Fast Polarity Switching, Non-Radioactive Drift Tube for the Miniaturization of Drift-Time Ion Mobility Spectrometer

Lingfeng Li, Hao Gu, Yanzhen Lv, Yunjing Zhang, Xingli He and Peng Li *

School of Electronic and Information Engineering, Soochow University, Suzhou 215006, China; lingfengli@suda.edu.cn (L.L.); hguguhao@stu.suda.edu.cn (H.G.); lvyanzhen110@gmail.com (Y.L.); yjzhang1223@suda.edu.cn (Y.Z.); hexingli@suda.edu.cn (X.H.)

* Correspondence: lipengsuda@suda.edu.cn; Tel.: +86-136-562-498-81

Contents

Figure S1. Photos of the drift tube device.

Figure S2. Example IMS spectrums of 5 narcotics: (**a**) 10 ng cocaine; (**b**) 50 ng methamphetamine; (**c**) 50 ng heroin; (**d**) 50 ng morphine; (**f**) 50 ng marijuana.

Figure S3. Example IMS spectrums of 6 explosives: (**a**) 50 ng black powder; (**b**) 50 ng PETN; (**c**) 100 ng HMX; (**d**) 50 ng RDX; (**f**) 100 ng HMTD; (**g**) 100 ng TATP.

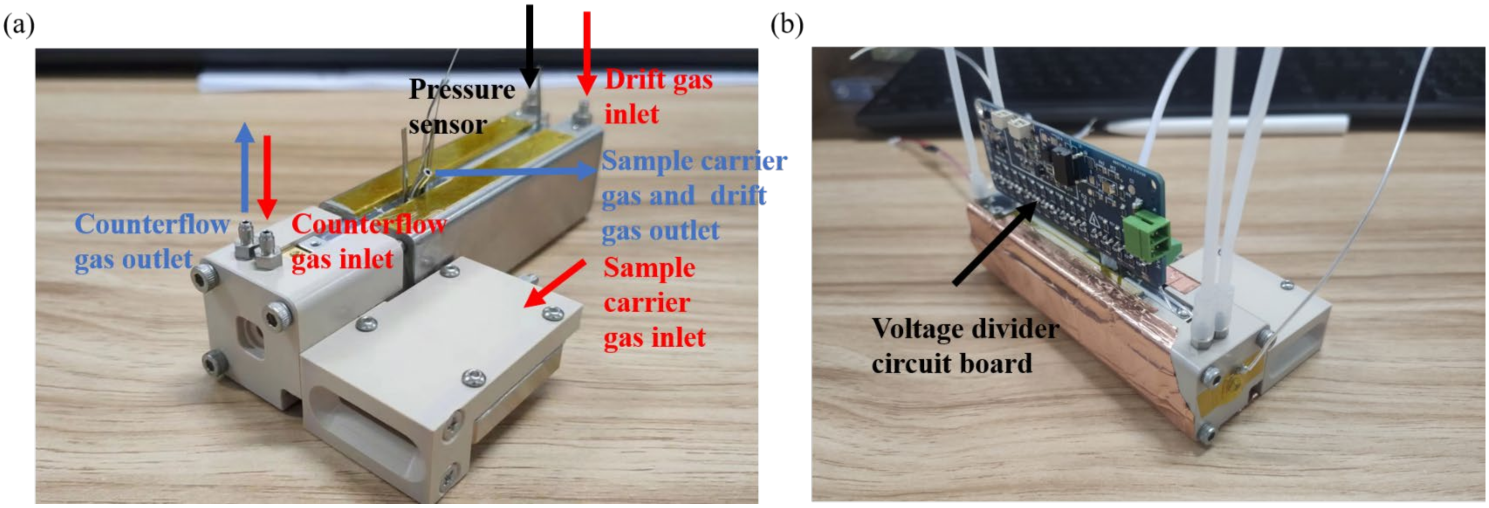

Figure S1. Photos of the drift tube device.

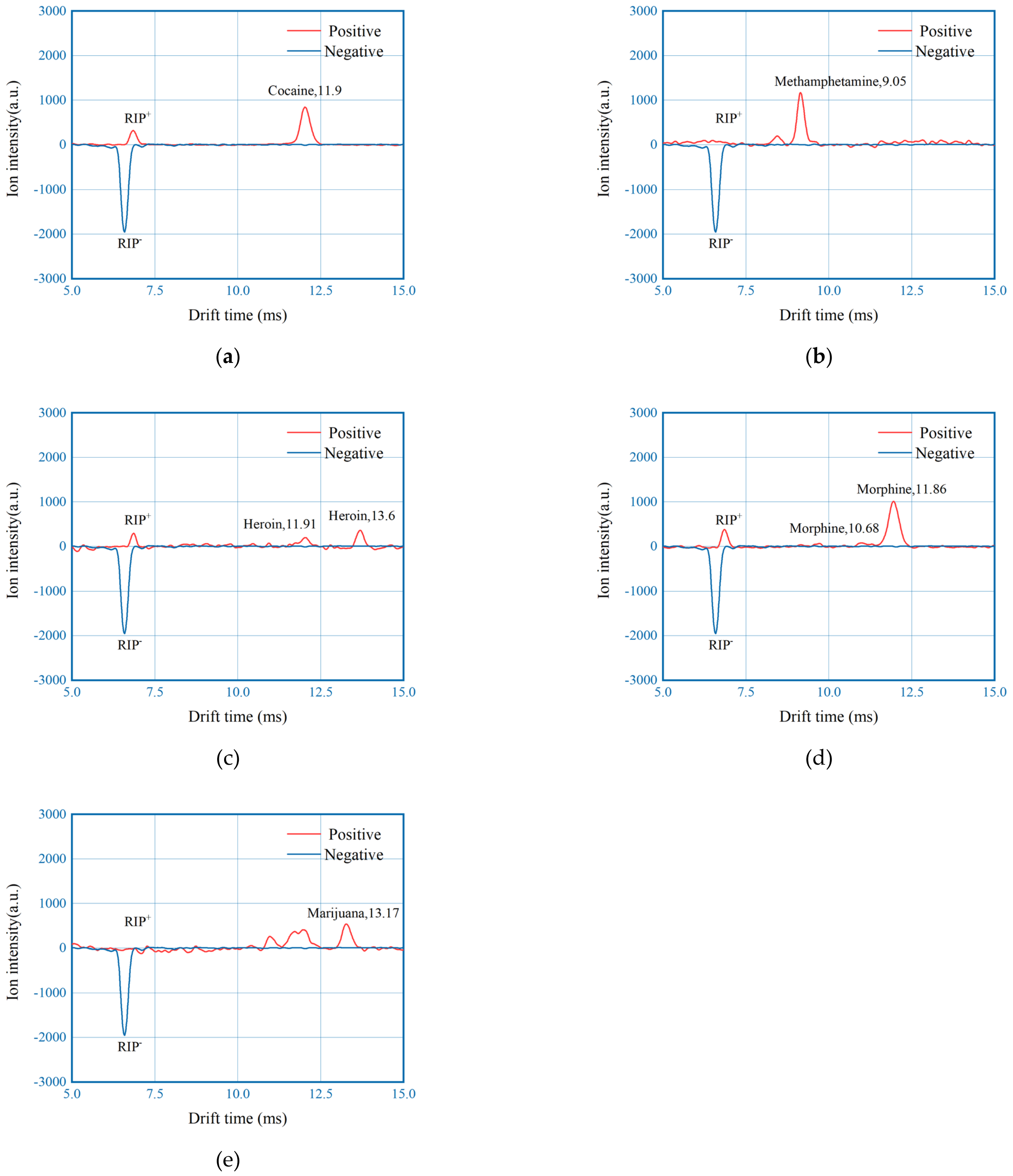

Figure S2. Example IMS spectrums of 5 narcotics: (**a**) 10 ng cocaine; (**b**) 50 ng methamphetamine; (**c**) 50 ng heroin; (**d**) 50 ng morphine; (**f**) 50 ng marijuana.

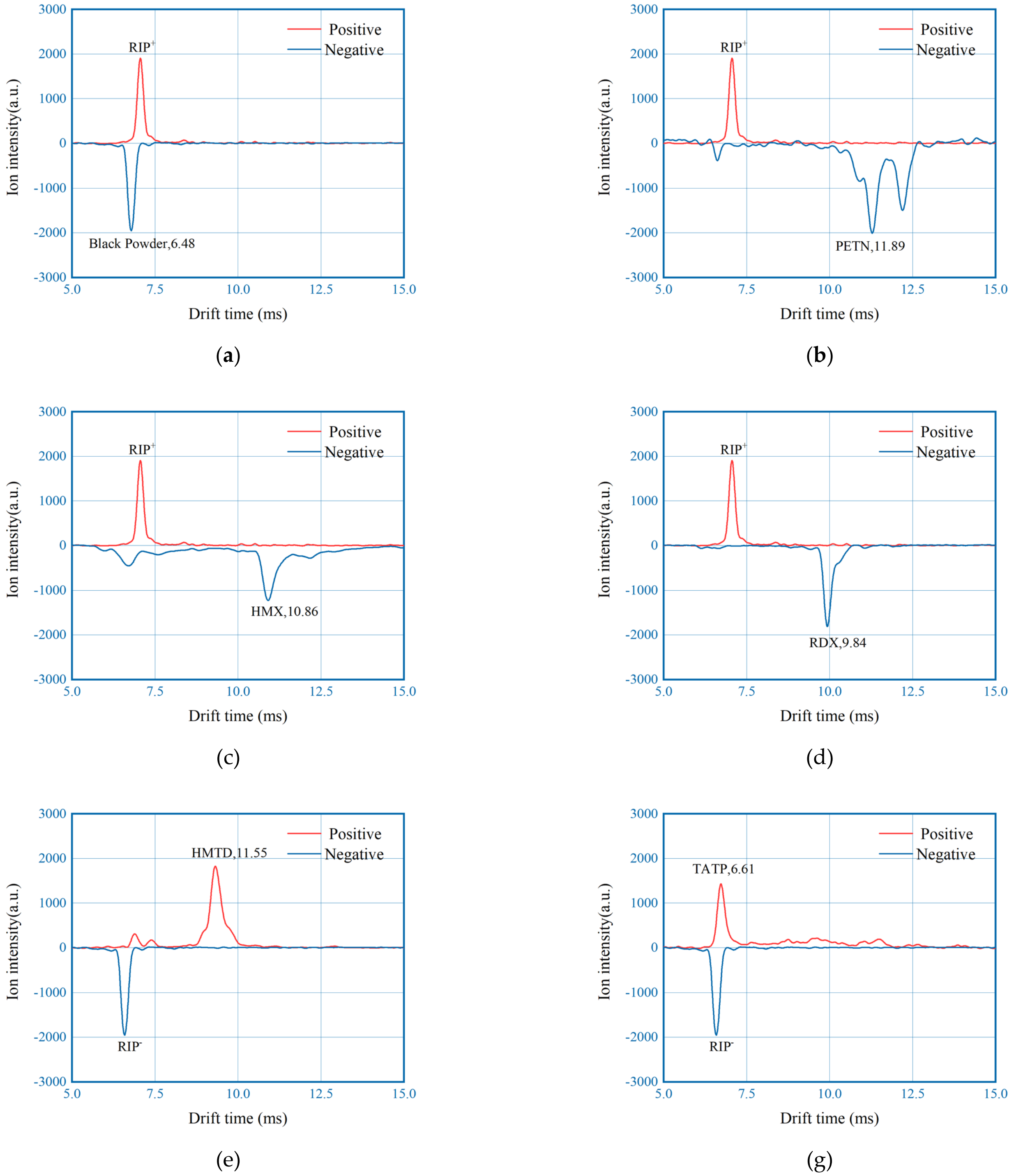

Figure S3. Example IMS spectrums of 6 explosives: (**a**) 50 ng black powder; (**b**) 50 ng PETN; (**c**) 100 ng HMX; (**d**) 50 ng RDX; (**f**) 100 ng HMTD; (**g**) 100 ng TATP.
